# Case report: catamenial hyperglycemia: the trigger of recurrent DKA in a female patient with three-year follow-up

**DOI:** 10.3389/fendo.2024.1305332

**Published:** 2024-02-20

**Authors:** Sumita Cholekho, Zuli Fan, Huiwen Tan

**Affiliations:** Department of Endocrinology and Metabolism, West China Hospital, Sichuan University, Chengdu, China

**Keywords:** catamenial hyperglycemia, diabetic ketoacidosis, menstrual cycle, luteal phase, diabetes mellitus

## Abstract

**Background:**

Catamenial hyperglycemia is a rare type of spontaneous, recurring Diabetic Ketoacidosis(DKA) in females during the luteal phase, most commonly observed in type 1 diabetes mellitus. Even with controlled serum glucose levels, adherence to a diabetic diet, medications, and in the absence of other common influencing factors such as infection, glucose levels tend to increase during the premenstrual period. This uncommon issue related to the menstrual cycle phase has not been extensively researched. Therefore, this study aims to diagnose catamenial hyperglycemia promptly and initiate early treatment to prevent complications.

**Case report:**

We presented a case of a 19-year-old girl who experienced recurrent DKA during the premenstrual period, without an apparent cause. She was admitted multiple times to various hospitals and sought consultations, undergoing numerous laboratory and imaging examinations, yet the etiology remained elusive. Ultimately, she received a diagnosis of catamenial diabetic hyperglycemia. To prevent recurrence of complications associated with catamenial hyperglycemia, we initiated a comprehensive approach which included continuous glucose monitoring, adherence to a strict diabetic diet, diabetic health education, regular exercise, timely medication administration, and increase in insulin dosage during the premenstrual period based on glucose levels.

**Conclusions:**

Although catamenial hyperglycemia is rare, it should be considered a cause of recurrent hyperglycemia in any postpubertal female to prevent complications. The specific underlying mechanisms responsible for catamenial hyperglycemia or DKA remain unidentified.

## Introduction

Diabetic ketoacidosis (DKA) represents the acute metabolic consequence of type 1 diabetes mellitus (T1DM), resulting from inadequate insulin levels in conjunction with an excess of regulatory hormones, including glucagon, catecholamines, cortisol, and growth hormone. Primary causes of DKA encompass infections, intercurrent illnesses, psychological stress, myocardial infarction, and inadequate adherence to therapy. Among these, infection stands out as the most prevalent precipitating factor for DKA, accounting for 30–50% of cases (1). DKA is a severe side effect of a metabolic condition that is characterized by hyperglycemia, and metabolic acidosis (2). It is associated with a severe inflammatory condition characterized by an elevation in proinflammatory cytokines (tumor necrosis factor-α and interleukin-β, -6, and -8), C-reactive protein, reactive oxygen species, and lipid peroxidation (3). Additionally, cardiovascular risk factors such as plasminogen activator inhibitor-1 and free fatty acids are elevated, even in the absence of a serious infection or cardiovascular pathology. Remarkably, within 24 hours of initiating insulin treatment and intravenous fluid hydration, pro-inflammatory cytokines return to normal levels (4). DKA may be induced by medications that influence carbohydrate metabolism, especially corticosteroids, thiazides, sympathomimetic drugs, and pentamidine (5). Antipsychotic medications have a negligible risk of triggering hyperglycemia and DKA. The currently available body of evidence provides an overall prevalence of DKA ranging from approximately 50 to 100 events per 1000, and mortality rates are approximately 5% for adult patients with T1DM (6). To effectively manage and lower the incidence of diabetic ketoacidosis (DKA), it is essential to identify its specific cause and make a definitive diagnosis. Pinpointing the exact reason behind recurrent DKA can often be challenging. One uncommon factor that has not received significant research attention is the phase of the menstrual cycle, known as catamenial DKA.

Throughout the menstrual cycle, a range of physiologic and hormonal changes take place, affecting the carbohydrate tolerance of certain diabetic women. The term “peri-menstrual DKA,” previously termed “catamenial DKA,” describes elevated blood sugar levels associated with the menstrual cycle. Catamenial illnesses, though widely reported, remain not entirely understood. The term ‘catamenial’ originates from the Greek word ‘katamenios,’ meaning monthly, referring to the menstrual period. Several medical conditions are recognized to have an association with the menstrual cycle, including epilepsy, pneumothorax, migraine, asthma, flares of rheumatoid arthritis, and neuropathy. Diabetic ketoacidosis (DKA) is another condition falling within this category (7). Nevertheless, the exact incidence and frequency of this association remain uncertain and have not yet been determined.Throughout the late luteal phase of their menstrual cycle, women experience hyperglycemia and may develop diabetic ketoacidosis (DKA). From the first case documented by Harrup and Mosenthal at John Hopkins Hospital in 1918 to the study conducted by Sennik et al. in 2010, only a small number of cases,of catamenial DKA/hyperglycemia have been reported (8). In 2017, the most recent case report was published, underscoring the intricacy of managing diabetes. This complexity highlights the necessity for closed-loop glucose control systems that can automatically adapt basal insulin patterns to meet the varying glucose management requirements during menstrual cycles. Implementing such systems could mitigate the risk of catamenial diabetic ketoacidosis, enhance overall glycemic control, and alleviate the psychological burden associated with type 1 diabetes (9). Therefore, this catamenial hyperglycemia is rare but needs to be focused on differential diagnosis. Here, we present a case of a 19-year-old female with a history of DM, who had recurrent DKA before menstruation. Due to the lack of additional aggravating situations, she suffered DKA, leading to catamenia. While, certain studies have demonstrated that blood sugar levels increase during the premenstrual or menstrual period, the specific underlying mechanisms responsible for catamenial hyperglycemia or DKA remain unidentified. Therefore, it is crucial to strive for preventive measures that can effectively avoid the recurrence of hyperglycemia or DKA episodes.

## Case presentation

This is a case of a 19-year-old young girl who was diagnosed with type 1 diabetes mellitus, 10 years ago at the age of 9. She was repeatedly presented in the emergency department with complaints of dry mouth, polyuria, polydipsia, nausea, vomiting, abdominal pain, and increased blood sugar levels. She was admitted to a local hospital in 2014, 2015, and 2016 for hyperglycemia; however, exact information about those hospital stays was not accessible. During that period, she was undergoing insulin treatment but inconsistently adhered to the prescribed regimen and did not consistently follow a diabetic diet.

Two years ago, despite denying any lapses in insulin administration and absence of infection, she experienced two admissions to our hospital.These admissions were due to diabetic ketoacidosis associated with type 1 diabetes mellitus, alongside complications including diabetic retinopathy, diabetic neurogenic bladder, hyponatremia, depression, and gastroenteritis. Six months ago, the patient presented to the emergency department with chief complaints of abnormal mental behavior and elevated blood glucose levels persisting for 7 days. She was subsequently admitted for 23 days and diagnosed with autoimmune encephalitis (viral encephalitis), epilepsy-like seizures, type 1 diabetic ketosis with gastrointestinal autonomic neuropathy, mental disorders secondary to physical diseases, chronic non-atrophic gastritis with constipation, gastrointestinal bleeding, increased intracranial pressure, and electrolyte disturbance. On physical examination T:36.5 C, P:98/min, RR:20/min, BP:158/98mmHg, height 165cm weight:60kg BMI:22kg/m2, dehydration was present.

Laboratory examination reports, as displayed in **Table 1**, indicated normal results for other routine examinations, lipid profile, and liver function. A complete lumbar puncture examination revealed that the initial pressure was greater than 200mmH_2_O, and the final pressure was 145mmH_2_0. Cerebrospinal fluid biochemistry showed glucose 8.47mmol/L and chlorine 131mmol/L. Cerebrospinal fluid culture and Gram stain results were negative for microorganism growth. A head CT conducted on December 3, 2022, yielded normal results. Based on her medical history, physical examination, and laboratory findings, it was suggested that the patient had diabetic ketoacidosis due to infection, which did not appear to be related to menstrual or hormonal imbalances. According to her menstrual history, she experienced menarche at age 11, had regular bleeding lasting 5 days without dysmenorrhea, and her last menstrual period occurred on November 10, 2022, with a regular cycle repeated every 40 days.

**Table 1:** Laboratory reports of patient (2020-2022).

Items	Normal Range	2020	2021	2022
**HbA1C (%)**	4.5-6.1	15	11.4	8.5
**Glucose(mmol/L)**	4-7.8	15.27	14.36	19.14
**PH**	7.35-7.45	7.344	7.363	7.373
**PO2(mmHg)**	80-100	93.9	96.9	171
**PCO2(mmHg)**	35-45	36.9	29.9	39.5
**Bicarbonate (mmol/L)**	22-27	11.7	16.6	20.6
**B Hydroxybutyric Acid(mmol/L)**	0.02-0.27	5.69	4.11	2.07
**Anion Gap(mmol/L)**	12-20	23.3	22.1	21.1
**Na(mmol/L)**	137-147	129.8	131	128.1
**K(mmol/L)**	3.5-4.5	4.86	3.37	3.37
**Urea(mmol/L)**	2.4-7.2	3.4	2.6	1.9
**Creatinine(umol/L)**	41-73	41	39	52
**WBC(/L)**	4.1-11*10^9^	5.44	5.6	11.11
**Neutrophil%** **Lymphocytes%** **Monocytes%** **Esinophil%** **Basophil%**	(40-75) (20-50) (3-10) (0.4-0.8) (0-1)	39.1 50.6 9 0.7 0.6	93.5 5.6 0.7 0 0.2	88.4 8.4 1.2 1.3 0.7
**Platelets(/L)**	100-300*10^9^	213	196	245
**Urine Ketones**	Nil	+4	+2	+3
**Lactate Dehydrogenase (Iu/L)**	105 - 333	131	166	345
**Procalcitonin(ng/ml)**	<0.046	<0.02	0.03	0.15
**IL_6(pg/ml)**	0-7	<1.5	<1.5	11.6
**C Reactive Protein(mg/L)**	<5	<1	1.44	4.22
**Prolactin(ng/mL)**	6-29.9	34.7	94	16.7
**Estradiol (pg/mL)**	32.69-201.04	42.2	14.9	83.59
**Progesterone(ng/mL)**	1.42 - 16.64	0.27	2.53	10.31
**LH(IU/L)**	0.8-15.5	7.7	0.71	2.73
**FSH (IU/L)**	1.3-23.4	4	3.2	2.7
**TSH(mU/L)**	0.27-4.2	1.7	1.42	1.7
**FT3 (pmol/L)**	3.6-7.5	4.12	4.83	4.29
**FT4(pmol/L)**	12-22	18.7	15.4	17.9
**PTH(pmo/L)**	1.6-6.9	3.61	-	-
**Glycated albumin value(%)**	9-14	49.86	–	-
**Glutamate decarboxylase(IU/mL)**	<10	1.08	-	2.56
**Insulin autoantibodies(COI)**	<1.1	0.47	–	1.09
**Islet cell antibody(COI)**	<1.1	0.04	-	0.19
**Anti Nuclear Antibody(ANA)**	negative	negative	–	negative

HbA1c, glycosylated hemoglobin A1C; PO2, partial pressure of oxygen; PCO2, partial pressure of carbon dioxide; Na, Sodium; K, Potassium; IL_6, Interleukin 6; LH, Luteinizing hormone(IU/L); WBC, White Blood Cell Count; FSH, Follicle-stimulating hormone; TSH, Thyroid stimulating hormone; FT3, free triiodothyronine; FT4, free thyroxine; PTH, parathyroid hormone.

-This means that the laboratory data were not obtained.

### Clinical findings

**Figure 1:**
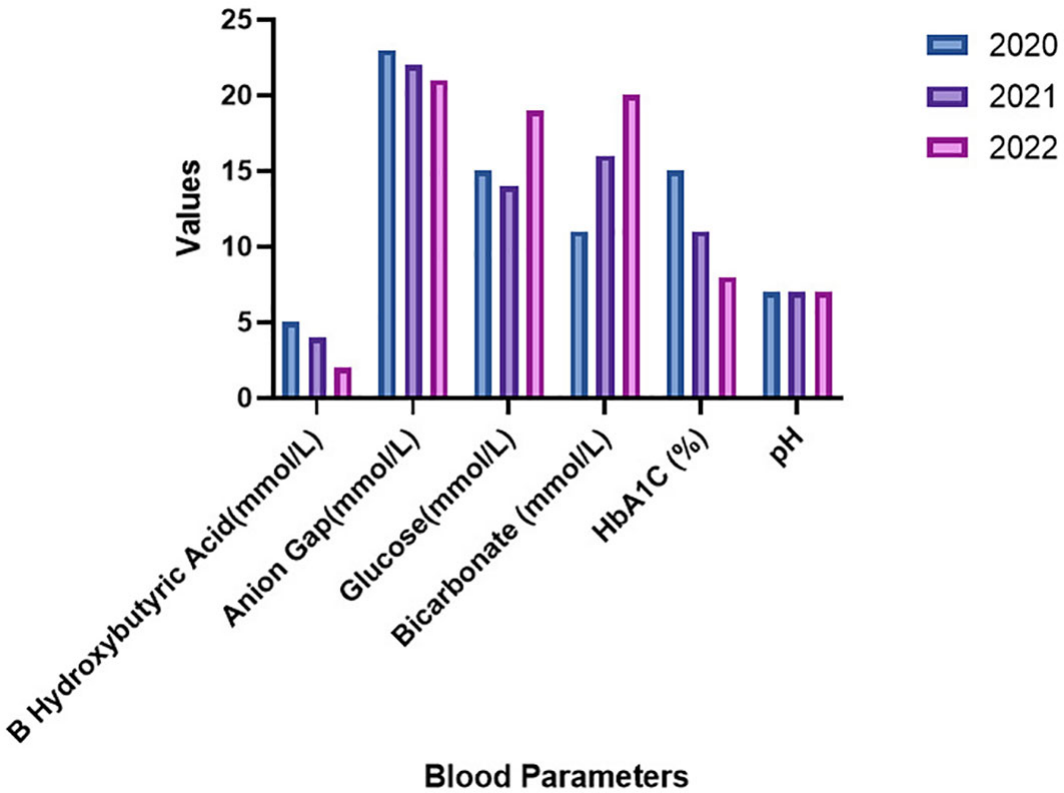
3 year time line from 2020- 2022 of hospitalization and blood parameters.

Laboratory examinations conducted during three consecutive hospitalizations in 2020 to 2022 are presented in **Table 1**. Other reports remained within normal limits. Notably, glucose levels in the emergency department exhibited a gradual increase with each admission, while HbA1c levels significantly decreased from 15% to 8.5% over three years. Moreover, the pH levels remained within the normal range, but urinary ketones consistently tested positive, ranging from 2 to 4, as illustrated in **Figure 1**.

### Differential diagnosis

As a diagnosed case of Type 1 Diabetes Mellitus (DM Type I), each presentation to the emergency department for this patient was marked by the first manifestation being diabetic ketoacidosis (DKA), which might be the underlying cause. Infections, particularly those in the urinary tract or chest, and occasionally viral encephalitis, have been identified as the most common precipitating factors. In this particular case, she has been admitted to our hospital two times due to DKA—twice attributed to catamenial factors.

### Treatment and follow-up

During each admission, the patient was managed according to the emergency department protocols involving insulin infusion, antibiotics, antiemetics, fluids, and electrolyte replacement. She started menstruating on the second or third day of each hospital stay. After her discharge, she was at regular follow-ups in the outpatient clinic. She was advised to continue taking daily basal and premeal insulin, and after raising the dosage during the premenstrual period, her blood sugar level was under control.

Clinical manifestations and laboratory examinations consistently aligned with a diagnosis of DKA related to her menstrual cycle. No other clear precipitant factors were identified during hospital visits, and complaints of hyperglycemia were not associated with recent infections or other symptoms. In addition,the insulin pump was working smoothly without any blockage.Comprehensive examinations, including complete blood count, liver function test, kidney function test, urinary pregnancy test, chest X-ray, urine examination, viral panel, ECG, and other imaging, all returned normal results.

The patient had a six-year history of depression with symptoms such as low mood, self-cut injuries on the left forearm, and poor sleep at night. She was prescribed sertraline 50 mg orally once a day and alprazolam 0.4 mg at night, which significantly improved her sleep disturbance and mood disorder. Gynecological consultations revealed no abnormalities. Notably, in all her admissions, she was in her premenstrual period, having regular cycles and being hospitalized approximately six times, twice in our hospital just before menstruation.

## Discussion

Women diagnosed with type 1 diabetes mellitus (T1DM) undergo fluctuations in their glycemic changeability due to variations in insulin receptor binding and affinity that are specifically linked to their menstrual cycle leading to Catamenial Hyperglycemia (10–12). Many studies has shown that compared to the early follicular phase, there is a higher likelihood of experiencing hyperglycemia during the periovulatory and early luteal phases. These patterns can differ among individuals and may even vary within the same person during successive menstrual cycles (13, 14). Therefore, patients can potentially gain the advantage of adjusting the insulin as per their different phases of menstrual cycle (15).

In our study, the patient diagnosed with type 1 diabetes mellitus, was admitted multiple times due to recurrent episodes of diabetic ketoacidosis (DKA). Ultimately, a diagnosis of catamenial DKA was established during her admission to our hospital. The evidence of this case suggested that the development of DKA was the casual occurrence of menstruation at the same time period of admission.

The menstrual cycle is divided into two distinct phases: the follicular phase (day one to day 13), comprising menstruation (day one to five) followed by ovulation (day 14), and the luteal phase (day 15 to 28). Two significant hormonal shifts occur during the menstrual cycle: a surge of estradiol before ovulation (days 10 to 15) and a decline in progesterone levels before menstruation (days 25 to 28). Progesterone plays a crucial role in governing the menstrual cycle, influencing pregnancy, and impacting factors such as sexuality, premenstrual syndrome, and dysmenorrhea. The menstrual cycle is regulated by various physiological components, with key contributors being FSH (follicle-stimulating hormone), LH (luteinizing hormone), their releasing factors in the hypothalamus, estrogen, and progesterone. The highest levels of progesterone in the bloodstream typically occur around 7 days after ovulation, followed by a gradual decrease to a very low level just before the onset of menstruation and on the first day of menstruation. Insulin resistance demonstrates a positive correlation with estradiol and progesterone levels, but it exhibits an inverse association with follicle-stimulating hormone (FSH) and sex hormone-binding globulin (16).

The precise mechanism behind catamenial hyperglycemia remains uncertain, but it is likely associated with decreased insulin sensitivity caused by elevated levels of circulating progesterone. Basic research in rats has indicated that progesterone can reduce glucose uptake in skeletal and adipose tissues (17). According to the findings of Cawood et al., approximately 67% of women with diabetes encountered fluctuations in their blood glucose levels during the premenstrual period (18). Widom et al. observed that premenstrual hyperglycemia in women with type 1 diabetes was linked to reduced insulin sensitivity, which was attributed to higher estrogen levels during the luteal phase. Additionally, they found that the deterioration in glucose uptake was associated with a more significant increase in estradiol levels from the follicular to the luteal phase (19). Goldner et al. with the help of a continuous glucose monitoring system, demonstrated that increased progesterone levels were the main reason for DKA and hyperglycemia during the luteal phase (14). The insulin receptor concentration exhibits a higher specific cell binding fraction during the follicular phase compared to the luteal phase. However, no such changes were observed in men or postmenopausal women during the same period. This indicates that sex hormones play a role and should be considered as one of the factors influencing insulin receptors (20).

Just before her subsequent menstruation, the patient in our research had a full sex-hormonal profile, which was normal. In this instance, the luteal phase progesterone and estrogen levels were not high as indicated.Consequently, based on those findings, it can be inferred that hormonal fluctuations alone may not be the sole factor contributing to catamenial DKA.

In various stages of the menstrual cycle, there have been observations of food cravings, such as binge eating and increased carbohydrate consumption, particularly during the luteal phase. These cravings are possibly influenced by higher progesterone levels, as seen in studies of women without diabetes (21, 22). While there have not been specific studies on women with diabetes regarding this matter, it is plausible to speculate that comparable dietary changes could occur, potentially contributing to the decline in glycemic control that some women with diabetes experience during their menstrual periods. Among young patients diagnosed with type 1 diabetes, approximately 20% of recurrent ketoacidosis cases might be influenced by psychological issues complicated by eating disorders (23).

In the present case, after being discharged from the hospital, she regularly followed-up in the outpatient clinics. However, the young girl was resistant to adhere the diabetic diet and expressed her desire for binge foods nearby her menstruation. In order to counterpoise her hyperglycemia and to overcome further episode of DKA, the patient was advised to increase the insulin dose 2 to 3 days prior her periods. Notably, she did not experienced any episodes of diabetic ketoacidosis (DKA) in the past 6 months. This positive outcome can be attributed to counseling on diabetic health education, exercise, and adherence to a diabetic diet. The medications prescribed for her sleep disturbance and anxiety, but not associated with increased blood glucose levels, effectively mitigated the stress-induced hyperglycemia.

Turning to the point of her viral encephalitis, there is no evidence to confirm that the recurrent ketoacidosis was related to opportunistic viral infection in this patient. The first two episodes of DKA did not have any evidence of encephalitis. Rather, outburst of her emotional state and eating disorder patterns during the period of menstruation had triggered uncontrolled glucose management and consequent ketoacidosis

We assume that the probable cause of opportunistic viral infections leading to viral encephalitis in patients with diabetes is immune deficiency. In supporting to this assumption, a previously published case report stated that opportunistic H1N1 viral influenza can trigger diabetic ketoacidosis in young women (24). However, there is no evidence to confirm that catamenial hyperglycemia was related to opportunistic viral infections in this patient. Interestingly,the first two episodes of DKA did not show any evidence of viral encephalitis; however, menstruation was present during each admission. Certainly, catamenial hyperglycemia followed by diabetic ketoacidosis versus viral encephalitis deserves further in-depth exploration.

DKA is a potentially the fatal disease yet preventable condition. Consequently, to avoid the recurrence of hyperglycemia or DKA, a proactive approach such as menstrual calendar, regular glucose monitoring with a glucometer, diabetic self-management, increasing the insulin dose before menstruation,diabetic diet, engaging in regular physical activity plays a important roles.

Additionaly, proper diabetic diet and exercise are key source for the management, avoidance of DKA and insuline resistance (25–27). A team of expert medically trained personals with indepth knowledge can be deployed to patient education, behavioral intervention, providing support for patients and families, improving patients’ access to medical providers, availability of extended access to telephone services and telemedicineto strengthen patient health education and awareness of ketoacidosis. In addition, public awareness campaigns focusing on education on the early signs of diabetes have been found to significantly reduce the frequency of DKA (28). Futhermore, in female patients with type 1 diabetes mellitus involves self-regulation by increasing the insulin dose by 1-2 units based on the blood glucose level few days prior to the expected pre-menstrual phase. As a result, every time a female patient with a known case of DM presents with hyperglycemia or DKA, a doctor should be wise enough to inquire about her menstrual state to identify the triggering factors due to the other reasons that were previously described.

In conclusion, challenges with blood sugar regulation can stem from various triggering factors, with the most common being infection and occasional causes such as hormonal fluctuations preceding menstruation. During the menstrual cycle, a natural increase in progesterone levels occurs during the luteal phase, contributing to increased insulin resistance thereby triggering catamenial hyperglycemia in females. However, the specific underlying mechanisms responsible for catamenial hyperglycemia or DKA remain unidentified.

### Strengths and limitations

Our case report incorporates distinctive clinical characteristics, allowing us to present a comprehensive clinical history encompassing present, past, personal, family, gynecological, and psychiatric consultations, as well as treatment details and outcomes. Notably, this is the first case report to document catamenial diabetic ketoacidosis associated with psychiatric problems. However, it is important to acknowledge the limitations of our study, including the absence of initial admission details from other hospitals and the lack of hormonal panel test results during different menstrual phases.

## Data availability statement

The original contributions presented in the study are included in the article/supplementary material. Further inquiries can be directed to the corresponding author.

## Ethics statement

Ethical approval was not required for the studies involving humans because this is a case report that does not involve any genetic or interventional therapy. The studies were conducted in accordance with the local legislation and institutional requirements. The participants provided their written informed consent to participate in this study. Written informed consent was obtained from the individual(s) for the publication of any potentially identifiable images or data included in this article.

## Author contributions

SC: Data curation, Formal analysis, Methodology, Writing – original draft. ZF: Investigation, Writing – original draft. HT: Conceptualization, Writing – review & editing, Data curation, Formal analysis.
